# Supplementary material for: Seminoma and Embryonal Carcinoma Footprints Identified by Analysis of Integrated Genome-Wide Epigenetic and Expression Profiles of Germ Cell Cancer Cell Lines
Source: PLoS One. 2014 Jun 2;9(6):e98330. doi: 10.1371/journal.pone.0098330 (PMC4041891; doi:10.1371/journal.pone.0098330)
Supplement: File S1 — ZIP file containing DMRforPairs output for significant regions. Please start from the html files. (ZIP) [file pone.0098330.s008.zip › figures/26963.pdf]

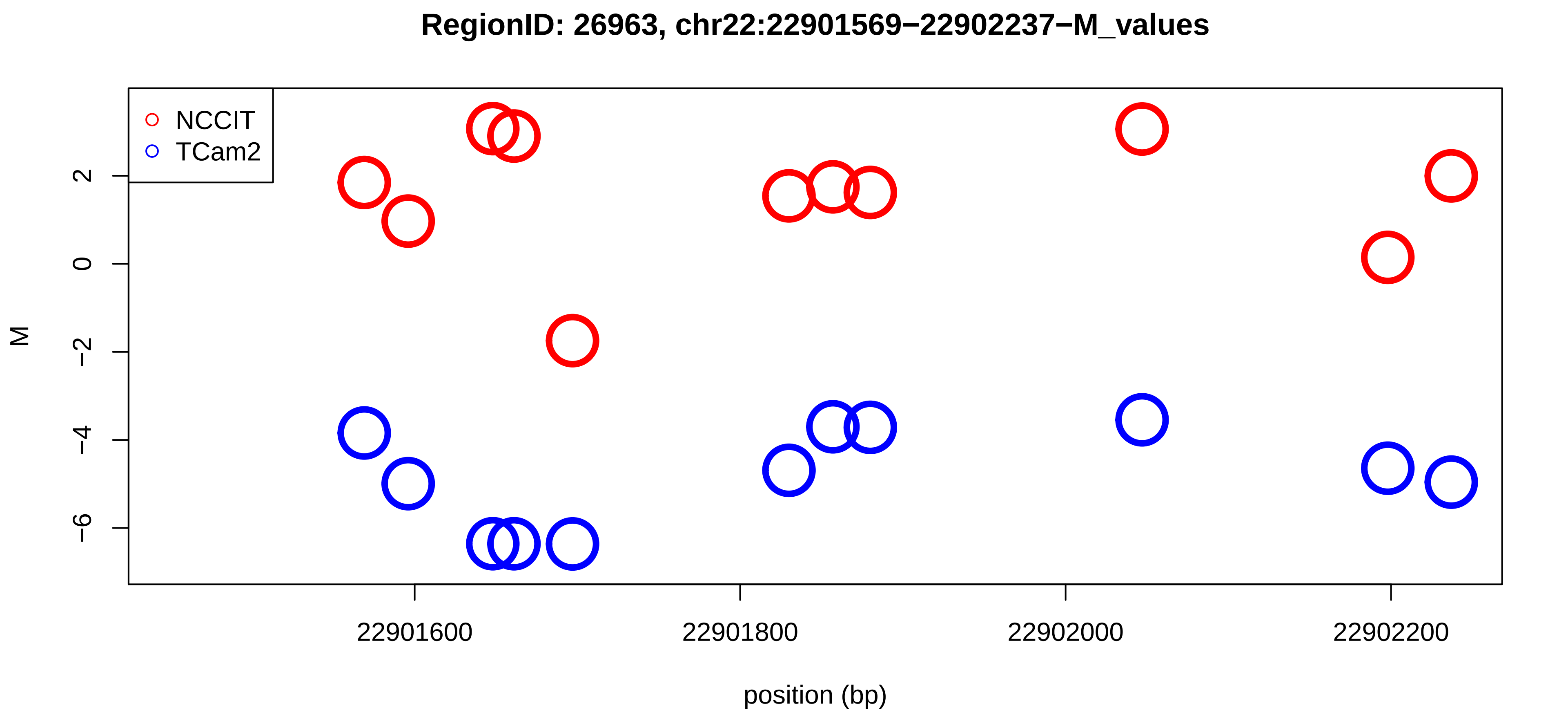
RegionID: 26963, chr22:22901569−22902237−M_values
NCCIT
TCam2
M
2
0
−2
−4
−6
22901600
22901800
22902000
22902200
position (bp)

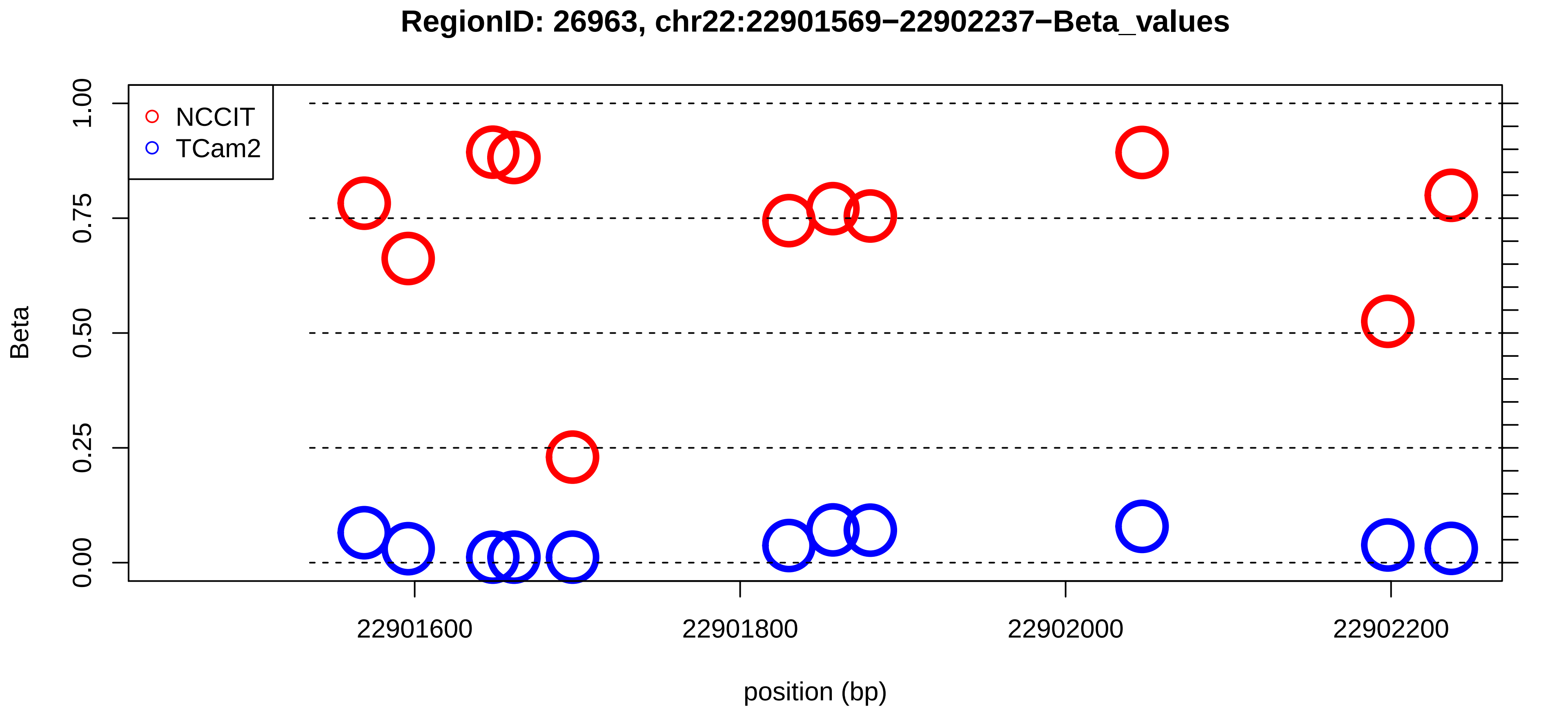
RegionID: 26963, chr22:22901569−22902237−Beta_values
NCCIT
TCam2
Beta
1.00
0.75
0.50
0.25
0.00
22901600
22901800
22902000
22902200
position (bp)

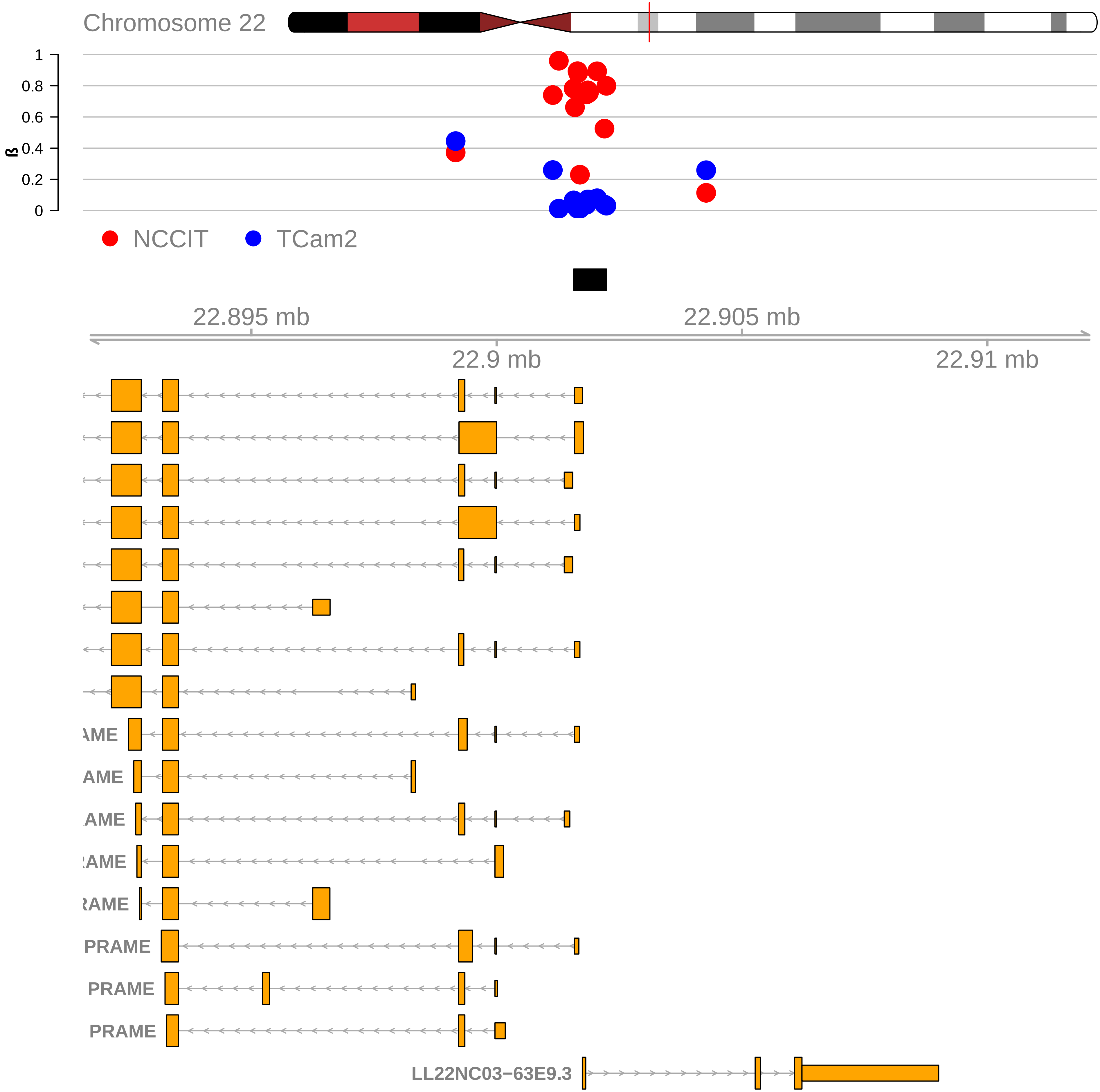

Chromosome 22
1
0.8
0.6
0.4
0.2
0
ß
NCCIT
TCam2
22.895 mb
22.905 mb
22.9 mb
22.91 mb
AME
AME
AME
RAME
RAME
PRAME
PRAME
PRAME
LL22NC03−63E9.3
